# Supplementary figures and images for: The AGE receptor, OST48 drives podocyte foot process effacement and basement membrane expansion (alters structural composition)
Source: Endocrinol Diabetes Metab. 2021 Jun 22;4(3):e00278. doi: 10.1002/edm2.278 (PMC8279619; doi:10.1002/edm2.278)

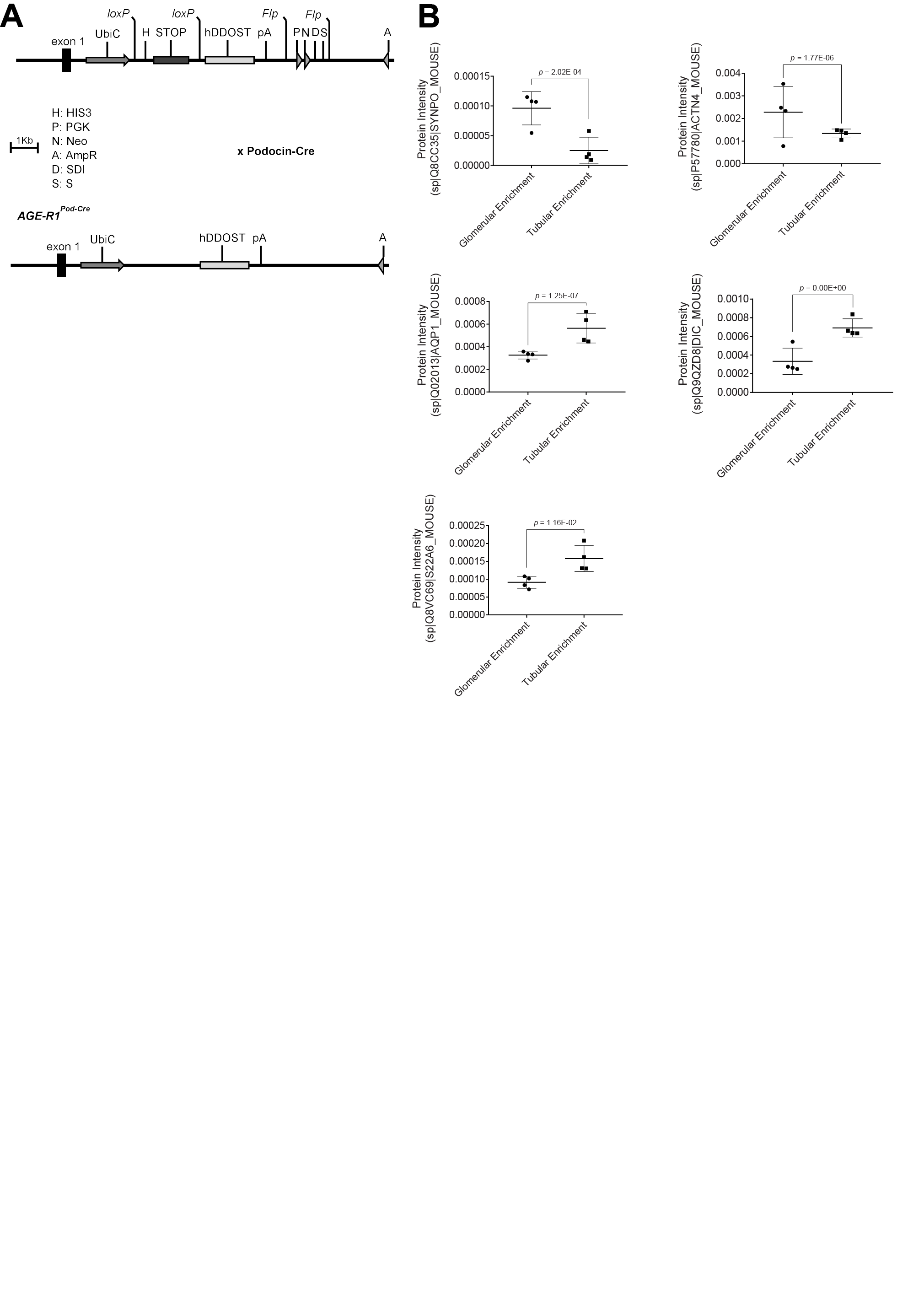

Supplement: Supplementary file 1 — Fig S1 [file EDM2-4-e00278-s001.png]

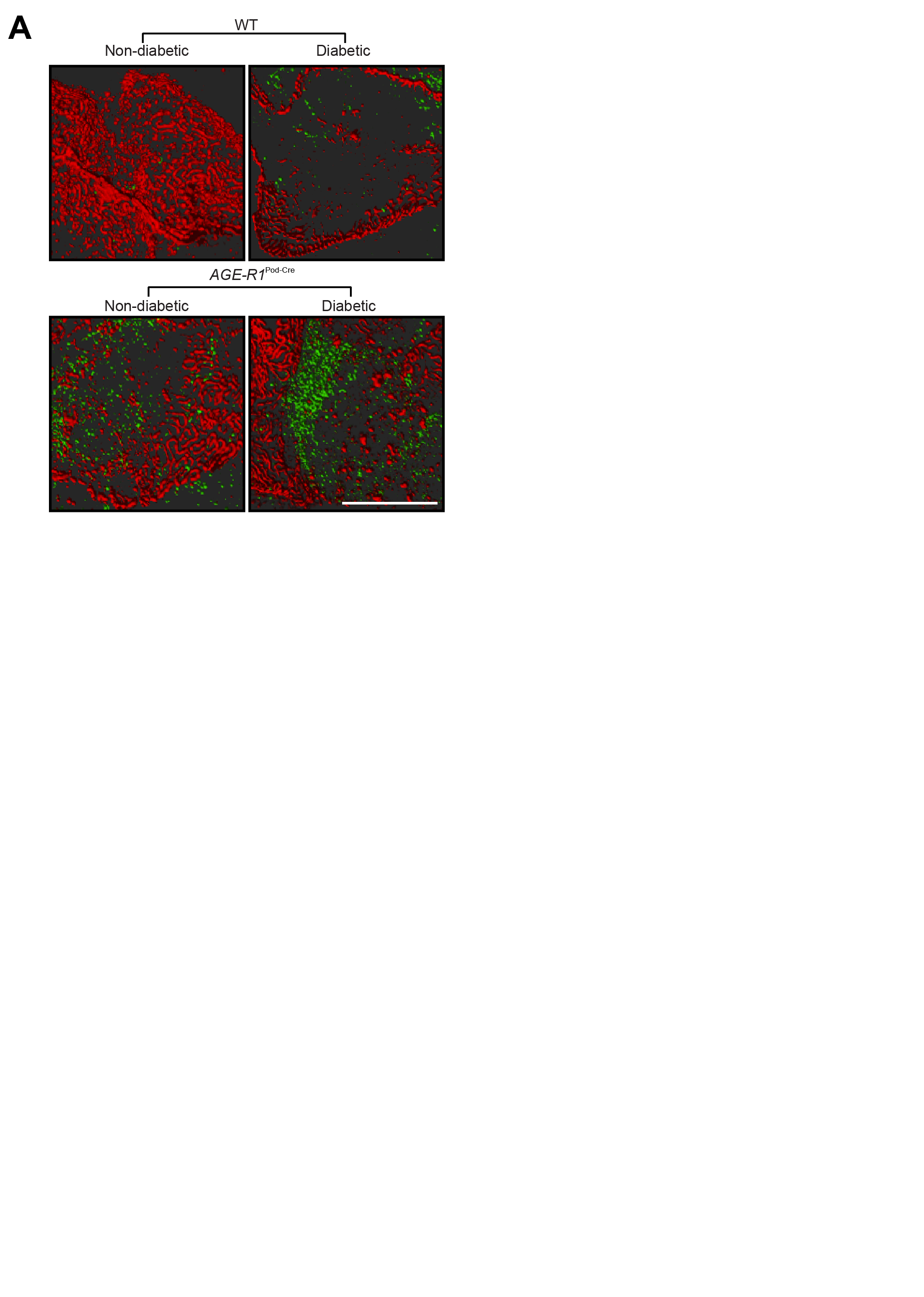

Supplement: Supplementary file 2 — Fig S2 [file EDM2-4-e00278-s005.png]

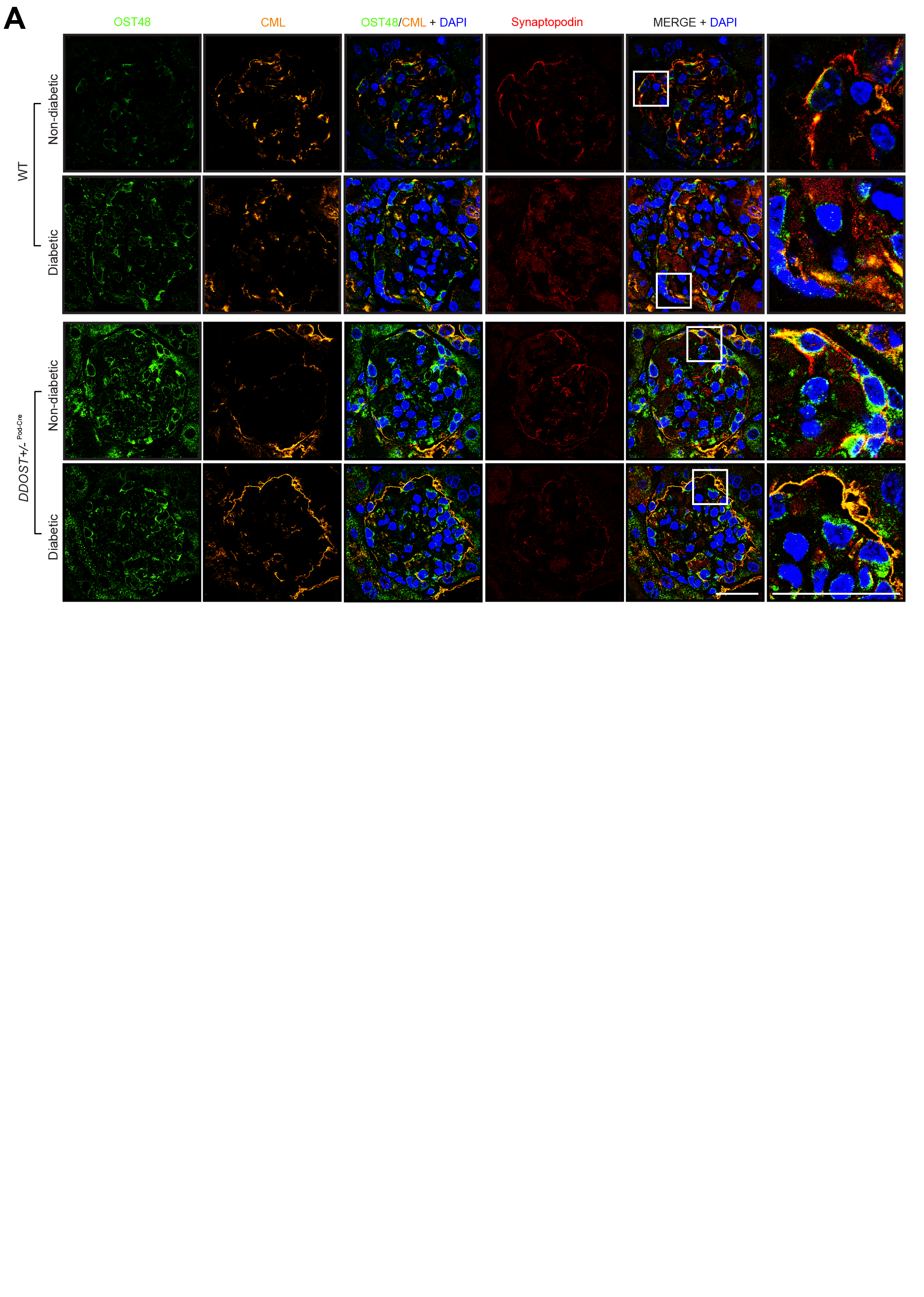

Supplement: Supplementary file 3 — Fig S3 [file EDM2-4-e00278-s002.png]
